# Spanish normative data of the Strengths and Difficulties Questionnaire in a community-based sample of adolescents: Datos normativos españoles del Cuestionario de capacidades y dificultades (SDQ) en una muestra comunitaria de adolescentes

**DOI:** 10.1016/j.ijchp.2022.100328

**Published:** 2022-08-29

**Authors:** Javier Ortuño-Sierra, Carla Sebastián-Enesco, Alicia Pérez-Albéniz, Beatriz Lucas-Molina, Eduardo Fonseca-Pedrero

**Affiliations:** 1 Department of Educational Sciences, University of La Rioja, C/ Luis de Ulloa, 2 (Edificio Vives) 26004, Logroño, Spain; 2 Department of Research and Psychology in Education, Complutense University of Madrid, C/ Campus de Somosaguas s/n 28223, Pozuelo de Alarcón (Madrid), Spain; 3 Department of Developmental and Education Psychology, University of Valencia, Av. Blasco Ibáñez, 21 46010 Valencia, Spain

**Keywords:** SDQ, Emotional-behavioral problems, Prosocial behavior, Normative data, Adolescence

## Abstract

*Background/Objective:* The Strengths and Difficulties Questionnaire self-report (SDQ-S) has been extensively used to assess mental health problems among children and adolescents. However, previous research has identified substantial age and country variation on its psychometric properties. The aim of this study was three-fold: i) to evaluate internal structure and measurement invariance of the Spanish version of the SDQ; ii) to analyze age and gender-specific effects on the SDQ subscales; and iii) to provide Spanish normative data for the entire age range of adolescence. *Method:* Data were derived from two representative samples of adolescents aged 14 to 19 years old, selected by stratified random cluster sampling years (*N* = 3378). *Results:* The reliability of the Total difficulties score was satisfactory, but some subscales showed lower levels of internal consistency. Confirmatory factor analysis supported the original five-factor model. Finally, results revealed that SDQ scores were influenced by the gender and the age of participants; thus, the normative banding scores and cut-off values were provided accordingly. *Conclusions:* This study validates the Spanish SDQ-S for the entire age range of adolescence. However, more cross-country and cross-age research is needed to better understand the inconsistent findings on SDQ reliability.

Mental health problems are common among children and adolescents worldwide (e.g., Achenbach et al., 2017). In Europe, mental disorders, including distinct emotional and behavioral problems, affect 5-20% of the child and adolescent population (Fonseca-Pedrero et al., 2016; UNICEF, 2021). Prevalence studies conducted with Spanish populations yielded a similar picture: around 10-21% of adolescents suffer from some sort of psychopathology, and around 21% of youth under 15 years of age are at risk of developing psychosocial disorders (Español-Martín et al., 2020; Ortuño-Sierra et al., 2018). Importantly, poor mental health produces significant negative consequences not only in the individual's life, but also in their families, school context, and the global community in general (e.g., Ross et al., 2020). It is worth noting that mental health is more than the absence of mental disorders and relies too on a series of attributes related to psychological well-being. Along this line, recent studies have identified the role of prosocial abilities in reducing the risk of developing mental disorders in children and adolescents (Abu-Akel et al., 2018; Fonseca-Pedrero et al., 2020).

Therefore, assessing children and adolescents’ mental health is crucial to promote public health policies. The Strengths and Difficulties Questionnaire (SDQ) (Goodman, 1997) is a well-established and widely used instrument for this purpose. It encompasses a self-report version as well as a parent and teacher version, and can be used both for clinical and research goals (Arman et al., 2013; Rodríguez-Hernández et al., 2012; Stone et al., 2010). Compared to other screening tools, the SDQ offers several advantages. First, it is a brief, multi-informant, easy-management instrument for use in a wide age range (from 2 to 19 years of age). Second, it assesses not only psychological difficulties (emotional, behavioral, and relationship problems), but also positive attributes (prosocial behavior). Third, the SDQ is free of charge, available online (www.sdqinfo.com), and has been validated in several populations (Garrido et al., 2018; He et al., 2013; Moriwaki & Kamio, 2014). However, the psychometric properties of the SDQ scores considerably vary across countries and age populations, and some key aspects related to evidence about its validity and reliability of the scores deserve further research.

First, some studies have revealed low values of reliability of the SDQ scores through Cronbach's alpha coefficient (*<* .60), especially in the subscales of Conduct problems and Peer problems (Goodman, 2001; Ruchkin et al., 2007). It is worth noting that the original format's response of the SDQ is a Likert type with three options. This could have contributed to the low levels of reliability previously found. Additionally, the use of Cronbach's Alpha has received different critics, as it implies that items are assumed to be continuous (e.g., Dunn et al., 2014). Thus, more recent work has opted for coefficients such as ordinal alpha or McDonald's Omega, reporting higher levels of reliability (Ortuño-Sierra, Fonseca-Pedrero, Paino, et al., 2015; Ortuño-Sierra et al., 2018; Stone et al., 2015), though, presumably, other factors may have contributed to these differences in reliability.

Another important psychometric consideration is the factor structure of the SDQ. Originally, the SDQ was developed to generate scores for five domains of psychological adjustment: Emotional symptoms, Conduct problems, Hyperactivity/Inattention problems, Peer problems, and Prosocial behavior (Goodman, 1997). However, subsequent empirical studies have yielded mixed results in the number of factors extracted. Whereas several works have provided support of the postulated five-dimension structure (He et al., 2013; Ortuño-Sierra, Fonseca-Pedrero, Paino, et al., 2015), others failed to replicate it, and proposed, instead, a three-factor solution consisting of an internalizing-problem dimension (combining the Emotional and Peer problems), an externalizing-problem dimension (combining the Conduct and Hyperactivity/Inattention problems), and the original positive factor (Prosocial behavior) (Goodman et al., 2010). Other authors, by contrast, claim that a bifactor solution can better contribute to understand the SDQ structure, as it might account for the high levels of comorbidity typically observed among behavioral and emotional problems (Kódor et al., 2013). In light of these inconsistent findings, some scholars have proposed that the number of factors could depend not only on the country (Essau et al., 2012; Ortuño-Sierra, Fonseca-Pedrero, Aritio-Solana, et al., 2015), but also on the age range (Van Roy et al., 2008).

Previous research on the use of SDQ in clinical routines has been conducted across countries, yet representative normative SDQ data are still limited to few populations and age ranges (Becker et al., 2018; Vugteveen et al., 2022). Only recently, a relevant study conducted with a Spanish sample of children and adolescents aged between 5 and 17, provided the normative data for the SDQ self-reported, teacher and parent versions (Español-Martín et al., 2020). Nonetheless, normative data in this study is only available for adolescents up to 17 years of age, leaving the 18- and 19-year-olds out of the scope. Also, age and gender comparisons were not established with the aim to justify the normative data. This is particularly important because previous research has shown that the SDQ scores frequently differ in boys and girls (Ortuño-Sierra et al., 2018), and may vary as a function of the individuals’ developmental period (Van Roy et al., 2008).

In order to fill the knowledge gaps concerning the performance of the SDQ in Spanish populations, the present study aimed at providing Spanish normative banding scores for the SDQ-S, based on data of a representative sample from the general population, and including the whole range of adolescence. In particular, we addressed the following goals: a) studying the internal consistency of the SDQ's scores; b) obtaining evidence about the internal structure of the SDQ; c) studying the measurement invariance of the SDQ by gender and age; d) analyzing the age and gender-specific effect on the Spanish SDQ subscales; and e) investigating and determining the normative banding scores and cut-off values of the SDQ for girls and boys as well as for different age ranges.

## Method

### Participants

We used two samples derived from two studies published elsewhere, conducted in 2016 and 2019, respectively. The samples were selected using stratified random cluster sampling, with the classroom as the sampling unit, from students of La Rioja (region located in northern Spain). The students belonged to different public and charter Secondary and Vocational Training Schools, and to different socio-economic groups. The layers were created as a function of the geographical zone and the educational stage.

Overall, the initial sample consisted of 3834 students. We removed those participants who presented a high score on the Oviedo Infrequency Response Scale (INF-OV) (more than 2 points) which indicated that their responses were not reliable (*n* = 250), and those participants who were older than 19 years old (*n* = 206). This resulted in a final sample of 3378 students, with 1561 males (46.2%), 1.804 (53.4%) females, and 13 (0.4%) other gender identity. The mean age was 15.8 years (*SD* = 1.26; age range = 14 to 19 years old). Due to the small number of 19-year-old participants, they were combined with the 18-year-old group. Distribution by age was: 14 years, *n* = 566; 15 years, *n* = 903; 16 years, *n* = 819; 17 years, *n* = 700; and 18 years, *n* = 390. The nationality distribution of the participants was predominantly represented by Spaniards (89.7%), followed by Romanian (2.5%) and Latin American (2.4%), an accurate reflection of the region population (INE, 2019).

### Instruments

*The Strengths and Difficulties Questionnaire* (SDQ), self-report version (Goodman, 1997). The SDQ is a tool used to measure emotional and behavioral difficulties and prosocial capacities in adolescents. It consists of a total of 25 items with a Likert-type response format (0 = *not true*, 1 = *somewhat true*, 2 = *certainly true*). SDQ items are grouped in five subscales (Hyperactivity, Conduct problems, Peer problems, Emotional symptoms, and Prosocial behavior). The psychometric properties of the Spanish version of the SDQ have been exanimated in previous studies (Ortuño-Sierra, Chocarro et al., 2015).

*The Oviedo Infrequency Scale* (INF-OV) (Fonseca-Pedrero, et al., 2009)*.* INF-OV was used to detect those participants who responded in a dishonest or random manner. The INF-OV is a self-report tool composed of 12 items rated on a 5-point Likert-type response scale (1 = *completely disagree*; 5 = *completely agree*).

### Procedure

Both studies were approved by The Research Ethics Committee of La Rioja (CEImLAR). The instruments were administered collectively via personal computers in classrooms of 10 to 30 students during a standard one-hour session and in rooms particularly prepared for this goal. For individuals under the age of 18, parents were asked to provide written informed consent. Participants were free to withdraw from the study at any time. No incentive was provided for their participation. Confidentiality was guaranteed to all participants.

### Data analyses

Given that the most significant age differences in the SDQ scores emerged between participants aged 16 years old or less and participants aged 17 years old or more (see below Age and Gender Effects), we, consequently, performed all analyses in relation to the following age groups: younger adolescents (14- to 16-year-olds) and older adolescents (17- to 18-year-olds). Crucially, these statistical differences correspond to the postulated stages of adolescence (i.e., early and late adolescence) (Salmero-Aro, 2011).

First, we examined the internal consistency of the SDQ items and subscales, and the Total difficulties score using the McDonald's Omega (Dunn et al., 2014).

Second, factor structure of the SDQ was examined through confirmatory factor analyses (CFA) following international guidelines (Ferrando et al., 2022). Several CFAs were conducted using the Diagonally Weighted Least Squares estimator and the polychoric correlation matrix. We tested different hypothetical factor models: a) the three-factor model with Internalizing and Externalizing problems and Prosocial capabilities as dimensions; b) a three-factor model with the inclusion of the correlated errors (CE) that were identified; c) the five-factor original model (Goodman, 1997); d) a five-factor model with CE; e) the five-factor model with two second-order factors (Goodman et al., 2010); f) the inclusion of the CE was also tested in model e; g) the bifactor model that includes a general factor and five dimensions (Kóbor et al., 2013); and h) finally, the bifactor model with the inclusion of the CE was also studied. Following Marsh et al. (2004), we set the criteria for acceptable model fit to RMSEA values below .08, together with CFI and TLI values above .90, and SRMR values lower than .08 as a good model fit.

Third, in order to test Measurement Invariance (MI) by gender and age, successive multigroup CFAs were conducted (Byrne, 2008). We performed multigroup comparisons through structural equation modelling under the measurement models (Byrne, 2008). First, we established the configural invariance model. Then, the strong invariance model, which contained cross-group equality constraints on all factor loadings and item thresholds, was calculated. Finally, factor means were fixed to zero in the first group and free in the other groups and scale factors were fixed to one in the first group and free in the other groups. Due to the limitations of the ∆χ2, we used the proposed ∆CFI criterion to determine if nested models were equivalent (Cheung & Rensvold, 2002).

Fourth, we explored the effect of age and gender in the SDQ scores. Due to the small number of participants reporting other-gender identity (*n* = 13), only individuals who described themselves as male or female were included in these analyses (*n* = 3355). We performed 2 × 5 ANOVA with the SDQ scores as outcomes, and gender (male, female) and age (14-, 15-, 16-, 17-, and 18-year-olds) as factors.

Finally, we computed the banding scores which allowed to identify clinical or “at risk” cases. For this purpose, we followed the same criteria used by Goodman in the original version of the SDQ (Goodman, 1997), and supported by empirical findings on the detection and prevalence of mental health problems (Achenbach et al., 2017; Goodman et al., 2000). On the basis that around 10% of the child and adolescent population display some kind of mental health problem and another 10% have a borderline problem, the threshold values designate scores above the 90th percentile in the “clinical” range, between the 80th and 90th percentile in the “at risk” range, and below the 80th percentile in the “non-clinical” category (Goodman et al., 1998; Goodman, 1997; Mellor, 2005). This was done for all subscales except for the Prosocial behavior, where scores equal or below the 10^th^ percentile and between the 10th and 20th percentiles were considered “clinical” and “at risk”, respectively.

Data analyses were performed using JASP (JASP Team, 2019).

## Results

### Descriptive statistics and internal consistency

The internal consistency of the Total difficulties score for the total sample was acceptable, ω = 0.74 (see Table S1, appendix A for descriptive statistics). The corresponding values for the subscales ranged from ω = 0.52 to ω = 0.71 (see Table S2, appendix A), which indicates that the internal consistency varied considerably across scales, with the lowest level of reliability for the Conduct problems.

### Evidence of validity based on internal structure

Goodness-of-fit indices for the three-factor baseline were poor (model a). The five-factor baseline model (model c) showed better fit but still did not reach the recommended cut-off points (see Table S3, appendix A). Substantial Modification Indices (MIs) (i.e., ≥ 25) were found for error correlation between items 2 (*restless*) and 10 (*fidgeting*), items 15 (*distracted*) and item 16 (*nervous or clingy*), item 7 and items 21 and 15 (*easily distracted*), items 19 (*bullied*) and 18 (*often lies or cheats*), and items 23 (*better with adults*) and 20 (*volunteers to help others*). We attended to correlated errors (CE) of those items that have similar content. Some of the items belong to the Hyperactive subscale, suggesting that this subscale could have overlapping items. Also, other CE suggest the possibility of overlapping between items from different subscales. Therefore, the model is far from being fully saturated.

After the inclusion of the CE, the five-factor solution (d) showed adequate goodness-of-fit indices. The modified three-factor solution (b) was still inadequate as well as the models with the inclusion of second-order factors (e and f). The bifactor model revealed adequate goodness-of-fit indices after the inclusion of the CE (h), however, some of the factor loadings were under .30. Thus, we decided to retain the five-factor solution with CE (d) as the most satisfactory model. All factor loadings were statistically significant in this model, ranging from .35 (item 23) to .78 (item 25). Correlations between factors were also all statistically significant, and ranged from -.31 (Emotional symptoms and Prosocial behavior) to .84 (Conduct problems and Hyperactivity).

### Measurement invariance of the SDQ scores across gender and age

We tested the measurement invariance of the five-factor model attending to gender and age. First, we tested whether the five-factor model with modifications showed a reasonably good fit to the data in each group. Then, we examined configural and strong MI (Table 1). A ΔCFI below .01 between the configural model and the metric model supported the hypothesis of weak MI across both gender and age. However, the ΔCFI was higher than .01 between the metric and the scalar models, confirming that scalar invariance was not supported.

**Table 1:** Goodness-of-fit indices for measurement invariance of the 5-factor model with modifications across gender and age

	*χ2*	*Df*	CFI	TLI	RMSEA (CI 90%)	SRMR	ΔCFI
**Gender**							
Male (*n* = 1561)	1725.38	262	.92	.91	.040 (.038-.042)	.04	
Female (*n* = 1804)	1629.50	262	.91	.91	.039 (.037-.042)	.06	
Configural Invariance	1698.35	405	.91	.90	.041 (.039-.043)	.05	
Metric Invariance	1612.13	625	.90	.90	.044 (.039-.049)	.07	-.01
Scalar Invariance	2532.45	715	.86	.86	.055 (.051-.059)	.09	+01
**Age**							
Younger (*n* = 2288)	1718.41	262	.91	.90	.041 (.039-.042)	.03	
Older 7-11 (*n* = 1090)	1695.52	262	.90	.90	.043 (.040-.046)	.04	
Configural Invariance	1746.62	405	.91	.90	.045 (.041-.049)	.06	
Metric Invariance	1789.40	625	.90	.90	.046 (.043-.049)	.06	-.01
Scalar Invariance	2618.11	715	.87	.86	.054 (.049-.058)	.08	+.01

*Note*. χ^2^ = Chi square; *df* = degrees of freedom; CFI = Comparative Fit Index; TLI = Tucker-Lewis Index; RMSEA = Root Mean Square Error of Approximation; CI = Confidence Interval; SRMR = Standardized Root Mean Square Residual; ΔCFI = Change in CFI.

### Age and gender effects

Regarding the gender differences, we found that girls scored higher than boys on the Total difficulties scale (*F*_(1,3355)_ = 57.49, *p* < .001, *η²* = .017), as well as on Emotional symptoms (*F*_(1,3355)_ = 339.02, *p* < .001, *η²* = .092), Peer problems (*F*_(1,3355)_ = 3.96, *p* = .047, *η²* = .001), and Prosocial behaviors (*F*_(1,3355)_ = 63.26, *p* < .001, *η²* = .019), whereas boys scored higher than girls on Conduct problems, *F*_(1,3355)_ = 33.76, *p* < .001, *η²* = .010. No gender differences were found on the Hyperactivity subscale.

The participants’ age had a significant effect on the Total difficulties score (*F*_(4,3355)_ = 2.98, *p* = .018, *η²* = .004), the Emotional symptoms (*F*_(4,3355)_ = 5.95, *p* < .001, *η²* = .007) and the Peer problems (*F*_(4,3355)_ = 3.26, *p* = .011, *η²* = .004) subscales, and a marginal effect on the Prosocial behavior subscale (*F*_(4,3355)_ = 1.96, *p* = .098). Post hoc testing using Bonferroni's correction revealed that 17-year-olds scored higher than 16-year-olds both in the Total difficulties score and the Emotional symptoms subscale, whereas in the Peer problems subscale, the difference emerged between the 18- and the 14-year-olds. No other age effect or interaction were found. Interestingly, most of these age effects coincide with the stages of the Spanish educational system (compulsory: up to 16 years of age, and post-compulsory: from 16 years of age onwards).

### Recommended bandings and cut-offs

Based upon the developmental and gender findings reported above, we calculated separate percentile ranks of raw values for two age groups, younger adolescents (14- to 16-year-olds), and older adolescents (17- to 18-year-olds) (Tables S4 and S5) as well as for boys and girls (Tables S6 and S7, appendix A). Threshold values for “non-clinical”, “at risk”, and “clinical” ranges were also calculated on the basis of the distributions of the SDQ's raw scores. We provide recommended banding scores for the total sample (Table 2) as well as gender- and age-specific bandings (Tables S4 to S7). In order to avoid an excessive number of false positive cases, we followed a criterion of sensitivity (i.e., identifying true positive rates) by ensuring that the percentage rate of “clinical” and “at risk” did not exceed 10% each, or 20% in total (except for the Prosocial behavior subscale where this criterion could not be met).

**Table 2:** *Banding scores for the Strengths and Difficulties Total difficulties score and subscales in the total sample* (*N* = 3365)

	Total		Emotional		Conduct		Hyper		Peer		Prosocial	
	Raw	PR	Raw	PR	Raw	PR	Raw	PR	Raw	PR	Raw	PR
	0-8	<40	0	9	0	22	0	3	0	31	0-3	0
	9	40	1	10	1	23	1	4	1	32	4	1
	10	48	2	24	2	48	2	11	2	62	5	3
	11	50	3	41	3	71	3	22	3	80	6	4
	12	56	4	56	4	85	4	37	4	90	7	10
	13	63	5	69	5	93	5	53	5	95	8	21
	14	70	6	79	6	97	6	70	6	98	9	40
	15	76	7	88	7	99	7	84	7-10	100	10	69
	16	81	8	94	8-10	100	8	92				
	17	85	9	97			9	98				
	18	89	10	>99			10	100				
	19	91										
	20	94										
	21	95										
	22	97										
	23-24	98										
	25	99										
	33-40	100										

Banding ranges												
NC	0-15 (80.5)		0-6 (87.2)		0-3 (84.8)		0-6 (83.2)		0-2 (80.0)		8-10 (79.8)	
AR	16-18 (10.5)		7 (6.2)		4 (8.0)		7 (8.8)		3 (9.7)		7 (11.1)	
CL	19-40 (9.0)		8-10 (6.6)		5-10 (7.2)		8-10 (8.0)		4-10 (10.3)		0-6 (9.1)	

*Note*. Hyper = Hyperactivity; Raw = Raw Score; PR = Percentile; NC = Non-Clinical; AR = At Risk; CL = Clinical.

## Discussion

This study aimed at establishing Spanish norms for the Strengths and Difficulties Questionnaire (SDQ), self-reported version, using data from two representative samples of adolescents aged 14 to 19 years old. We examined the reliability of the SDQ's subscales and the Total difficulties score and tested the factor structure of the SDQ by exploring the different hypothesized dimensional models. Additionally, we were interested in developing generalized, as well as gender- and age-specific, norms for research goals and clinical practice.

The examination of the reliability of the scores revealed that the Total difficulties score, as well as the Emotional symptoms and the Hyperactivity subscales reached a sufficient level of internal consistency of the scores. However, Peer problems, Prosocial behavior, and Conduct problems were less than satisfactory, with the lowest values related to the latter subscale. Previous studies also show weaknesses concerning the reliability of some SDQ subscales in different populations from Australia, Japan, Netherlands, and Spain (Becker et al., 2018; Mellor & Stokes, 2007; Moriwaki & Kamio, 2014; Ortuño-Sierra, Fonseca-Pedrero, Aritio-Solana et al., 2015). Stone et al. (2010) argued that the low correlations among scale items may be due to the fact that some reverse-worded items do not always reflect the same construct as the remaining items of a given subscale. Moreover, the SDQ comprises only five items to evaluate complex and multicausal psychosocial phenomena. Thus, items may assess different but related issues, resulting in less homogeneous scales. Interestingly, recent studies suggest that beyond the features of the SDQ itself, low levels of consistency are due to the adolescents being only partially aware of their own difficulties and strengths (Filippi et al., 2020). More research is needed to further understand the role of the adolescents’ limited metacognitive skills in these findings. One promising way is to combine the use of the SDQ with its impact supplement that asks, in a more explicit manner, if the adolescents are experiencing any problems in different areas of their lives.

Regarding the latent structure of the SDQ, the findings support the original proposed five-factor structure among Spanish adolescents up to the age of 19 (Goodman, 1997) rather than the other solutions tested, namely, the broader three-factor model consisting of internalizing and externalizing problems together with the prosocial factor (Goodman et al., 2010) and the hierarchical bifactor solution (Kódor et al., 2013). Simplifying the SDQ factor structure also produced a less accurate model fit when data was split by gender and age group. Previous research, albeit scarce, shows considerable variations in the latent structure as a function of the country (Ortuño-Sierra, Fonseca-Pedrero, Aritio-Solana, et al., 2015). Regarding the Spanish self-reported version of the SDQ, Español-Martin et al. (2020) found a better fit of the five-factor over the three-factor model among Spanish children aged 5 to 17 years old. Together with our findings, this indicates that the original structure of the SDQ is preferred when evaluating the whole age range of Spanish children and adolescents from the general population.

Similar to previous research, girls and boys differed in their self-reported levels of psychosocial adjustment. Girls evaluated themselves as more prosocial and reported fewer conduct problems than did boys. However, they were more likely to report problems in general (Total difficulties score), emotional symptoms in particular, when compared to boys. These findings are not only in line with several normative studies (Becker et al., 2018; Mellor, 2005; Vugteveen et al., 2022) but are also partially consistent with clinical research showing that girls display more internalizing problems and boys present more externalizing problems (e.g., Achenbach et al., 2017). In addition, girls in the present study reported higher levels of peer-related problems than boys. Previous findings are less consistent in this respect: whereas some studies showed that boys were more likely to report problems with their peers (Yao et al., 2009), others did not find gender differences (Becker et al., 2018). Some studies have found that the SDQ Peer problem subscale correlated moderately with scales assessing internalizing problems such as the CBCL internalizing subscales (Stone et al., 2010) or the withdrawal/depressed YSL subscale (Yao et al., 2009). It is thus plausible that youth may interpret some of the Peer problem's items as more related to loneliness, for instance (e.g., tends to play alone, has at least one good friend) than behavioral problems.

Regarding age differences, we found that Total difficulties increased with age, as did Emotional symptoms and Peer-related problems. Interestingly, the levels of Prosocial Behavior also tended to be higher in older than younger adolescents. These findings fit the general expectations of adolescent development characterized by an increasing awareness of their own skills and limitations, along with the emergence of more complex and adequate prosocial responses (Malti & Dys, 2018; Rodríguez-Fernández et al., 2016). However, both self-reported Hyperactivity and Conduct problems did not change over time. Previous findings are less consistent about this. Some studies reported an increasing tendency with age (Chinese adolescents: Yao et al., 2009), whereas others indicated the opposite tendency (Iranian adolescents: Arman et al., 2013) or found no differences (Sri Lanka adoelscents: Prior et al., 2005). Although any interpretation should be taken cautiously given the cross-cultural nature of the data, one possible explanation may be related once again to the progressive acquisition of metacognitive skills during adolescence. This results in advances not only in the ability to identify one's own problems, but more importantly, in the tendency to present oneself in a culturally appropriate fashion which may lead to some youth only partially reporting existing problems.

Some limitations of the present study must be acknowledged. First, the findings were based on a self-report by adolescents. This presents a series of well-known problems related to social desirability and metacognitive skills, especially when evaluating adolescents. Second, even though our findings support the five-factor structure for the SDQ, goodness-of-fit indices were not always acceptable. This may suggest that some of the items from the Spanish version should be further examined and if needed, reworded, to increase the reliability of some of the subscales. Finally, the data of this study was derived from a general population with a low frequency of adolescents presenting mental health problems. This might have limited the diagnostic accuracy of some of the SDQ's subscales. A next step should be to explore the diagnostic predictions with the Spanish version of the SDQ in clinical population. Also, future research could focus on network analysis with the aim to obtain a more in depth comprehension of etiological mechanisms as well as protective factors. Network analysis could be relevant in order to reduce the limitations of models (e.g., medical model) based on common latent causes (Fonseca-Pedrero, 2017). Moreover, the inclusion of item response theory (IRT) in future research may allow to study the relationship between latent traits (e.g., unobservable characteristics or attributes) and their manifestations (e.g., responses or performance) and, thus, establishing the individual's position on a given continuum.

## Conclusions

The use of a simple and brief screening instrument such as the SDQ may help to better understand mental health problems during adolescence, and to investigate the observed gender, age and country influences. In addition, reliable and valid assessment of mental health among young people is essential, as it enables early detection and identification of clinical and at-risk cases, which in turn may help to guide the psychological treatment at a critical stage of human development such as adolescence (Fonseca-Pedrero et al., 2021). This study validates the self-reported version of the Spanish SDQ for the whole age range of adolescence, and provides the normative data and threshold values for boys and girls, and younger and older adolescents, in order to ensure an adequate interpretation of individuals’ raw values.

## Funding

This research was funded by the “Convocatoria 2015 de Ayudas Fundación BBVA a Investigadores y Creadores Culturales”, the “Ayudas Fundación BBVA a Equipos de Investigación Científica 2017”, the “Beca Leonardo a Investigadores y Creadores Culturales 2020 de la Fundación BBVA” and FEDER in the PO FEDER of La Rioja 2014-2020 (SRS 6FRSABC026).

## Declaration of Competing Interest

The authors have declared that there are no conflicts of interest in relation to this study**.**

## Appendix Supplementary materials

Image, application 1

## References

[bib0001] Abu-Akel A., Baxendale L., Mohr C., Sullivan S. (2018). The association between schizotypal traits and social functioning in adolescents from the general population. Psychiatry Research.

[bib0002] Achenbach T.M., Ivanova M.Y., Rescorla L.A. (2017). Empirically based assessment and taxonomy of psychopathology for ages 1½–90+ years: Developmental, multi-informant, and multicultural findings. Comprehensive Psychiatry.

[bib0003] Arman S., Amel A.K., Maracy M.R. (2013). Comparison of parent adolescent scores on Strengths and Difficulties Questionnaire. Journal of Research in Medical Sciences : The Official Journal of Isfahan University of Medical Sciences.

[bib0004] Becker A., Wang B., Kunze B., Otto C., Schlack R., Hölling H., Ravens-Sieberer U., Klasen F., Rogge J., Isensee C., Rothenberger A. (2018). Normative data of the self-report version of the German strengths and difficulties questionnaire in an epidemiological setting. Zeitschrift Fur Kinder- Und Jugendpsychiatrie Und Psychotherapie.

[bib0005] Byrne B. (2008). Testing for multigroup equivalence of a measuring instrument: A walk through the process. Psicothema.

[bib0006] Cheung G.W., Rensvold R.B. (2002). Evaluating goodness-of-fit indexes for testing measurement invariance. Structural Equation Modeling: A Multidisciplinary Journal.

[bib0007] Dunn T.J., Baguley T., Brunsden V. (2014). From alpha to omega: A practical solution to the pervasive problem of internal consistency estimation. British Journal of Psychology.

[bib0008] Español-Martín G., Pagerols M., Prat R., Rivas C., Sixto L., Valero S., Artigas M.S., Ribasés M., Ramos-Quiroga J.A., Casas M., Bosch R. (2020). Strengths and difficulties questionnaire: psychometric properties and normative data for Spanish 5- to 17-year-olds. Assessment.

[bib0009] Essau C., Olaya B., Anastassiou-Hadjicharalambous X., Pauli G., Gilvarry C., Bray D., O'callaghan J., Ollendick T.H. (2012). Psychometric properties of the Strength and Difficulties Questionnaire from five European countries. International Journal of Methods in Psychiatric Research.

[bib0010] Ferrando P.J., Lorenzo-Seva U., Hernández-Dorado A., Muñiz J. (2022). Decalogue for the factor analysis of test items. Psicothema.

[bib0011] Filippi R., Ceccolini A., Periche-Tomas E., Bright P. (2020). Developmental trajectories of metacognitive processing and executive function from childhood to older age. Quarterly Journal of Experimental Psychology.

[bib0012] Fonseca-Pedrero E. (2017). Network analysis: A new way of understanding psychopathology?. Revista de Psiquiatría y Salud Mental.

[bib0013] Fonseca-Pedrero E., Díez-Gómez A., de la Barrera U., Sebastián-Enesco C., Ortuño-Sierra J., Montoya-Castilla I., Lucas-Molina B., Inchausti F., Perez-Albeniz A. (2020). Suicidal behaviour in adolescents: A network analysis. Revista de Psiquiatrí́a y Salud Mental.

[bib0014] Fonseca-Pedrero E., Gooding D.C., Ortuño-Sierra J., Pflum M., Paino M., Muñiz J. (2016). Classifying risk status of non-clinical adolescents using psychometric indicators for psychosis spectrum disorders. Psychiatry Research.

[bib0015] Fonseca-Pedrero E., Lemos-Giráldez S., Paino M., Villazón-García U., Muñiz J. (2009). Validation of the Schizotypal Personality Questionnaire Brief form in adolescents. Schizophrenia Research.

[bib0016] ໿Fonseca-Pedrero E., Pérez-Álvarez M., Al-Halabí S., Inchausti F., López-Navarro E.R., Muñiz J., Lucas-Molina B., Pérez-Albéniz A., Baños Rivera R., Cano-Vindel A., Gimeno-Peón A., Prado-Abril J., González-Menéndez A., Valero A.V, Priede A., González-Blanch C., Ruiz-Rodríguez P., Moriana J.A., Gómez L.E., Montoya-Castilla I. (2021). Tratamientos Psicológicos Empíricamente Apoyados Para la Infancia y Adolescencia: Estado de la Cuestión [Empirically Supported Psychological Treatments for Children and Adolescents: State of the Art]. Psicothema.

[bib0017] Garrido L.E., Barrada J.R., Aguasvivas J.A., Martínez-Molina A., Arias V.B., Golino H.F., Legaz E., Ferrís G., Rojo-Moreno L. (2018). Is small still beautiful for the strengths and difficulties questionnaire? Novel findings using exploratory structural equation modeling. Assessment.

[bib0018] Goodman A., Lamping D.L., Ploubidis G.B. (2010). When to use broader internalising and externalising subscales instead of the hypothesised five subscales on the strengths and difficulties questionnaire (SDQ): Data from british parents, teachers and children. Journal of Abnormal Child Psychology.

[bib0019] Goodman R. (1997). The strengths and difficulties questionnaire: A research note. Journal of Child Psychology and Psychiatry and Allied Disciplines.

[bib0020] Goodman R. (2001). Psychometric properties of the strengths and difficulties questionnaire. Journal of the American Academy of Child & Adolescent Psychiatry.

[bib0021] Goodman R., Ford T., Simmons H., Gatward R., Meltzer H. (2000). Using the Strengths and Difficulties Questionnaire (SDQ) to screen for child psychiatric disorders in a community sample. British Journal of Psychiatry.

[bib0022] Goodman R., Meltzer H., Bailey V. (1998). The strengths and difficulties questionnaire: A pilot study on the validity of the self-report version. European Child and Adolescent Psychiatry.

[bib0023] He J.P., Burstein M., Schmitz A., Merikangas K.R. (2013). The strengths and difficulties questionnaire (SDQ): The factor structure and scale validation in U.S. Adolescents. Journal of Abnormal Child Psychology.

[bib0024] INE (2019). https://www.ine.es/dyngs/INEbase/es/operacion.htm?c=Estadistica_C&cid=1254736177011&menu=resultados&idp=1254734710990.

[bib0025] JASP Team. (2019). JASP (Version 0.11.1.0) [Computer software].

[bib0026] Kóbor A., Takács Á., Urbán R. (2013). The bifactor model of the strengths and difficulties questionnaire. European Journal of Psychological Assessment.

[bib0027] Malti T., Dys S.P. (2018). From being nice to being kind: development of prosocial behaviors. Current Opinion in Psychology.

[bib0028] Marsh H.W., Hau K.T., Wen Z. (2004). In search of golden rules: Comment on hypothesis-testing approaches to setting cutoff values for fit indexes and dangers in overgeneralizing Hu and Bentler's (1999) findings. Structural Equation Modeling.

[bib0029] Mellor D. (2005). Normative data for the Strengths and Difficulties Questionnaire in Australia. Australian Psychologist.

[bib0030] Mellor D., Stokes M. (2007). The factor structure of the strengths and difficulties questionnaire. European Journal of Psychological Assessment.

[bib0031] Moriwaki A., Kamio Y. (2014). Normative data and psychometric properties of the strengths and difficulties questionnaire among Japanese school-aged children. Child and Adolescent Psychiatry and Mental Health.

[bib0032] Ortuño-Sierra J., Aritio-Solana R., Fonseca-Pedrero E. (2018). Mental health difficulties in children and adolescents: The study of the SDQ in the Spanish National Health Survey 2011–2012. Psychiatry Research.

[bib0033] Ortuño-Sierra J., Chocarro E., Fonseca-Pedrero E., Sastre-Riba S., Muñiz J. (2015). The assessment of emotional and Behavioural problems: Internal structure of The Strengths and Difficulties Questionnaire. International Journal of Clinical and Health Psychology.

[bib0034] Ortuño-Sierra J., Fonseca-Pedrero E., Aritio-Solana R., Velasco A.M., de Luis E.C., Schumann G., Cattrell A., Flor H., Nees F., Banaschewski T., Bokde A., Whelan R., Buechel C., Bromberg U., Conrod P., Frouin V., Papadopoulos D., Gallinat J., Garavan H., Lawrence C. (2015). New evidence of factor structure and measurement invariance of the SDQ across five European nations. European Child and Adolescent Psychiatry.

[bib0035] Ortuño-Sierra J., Fonseca-Pedrero E., Paino M., Sastre i Riba S., Muñiz J. (2015). Screening mental health problems during adolescence: Psychometric properties of the Spanish version of the Strengths and Difficulties Questionnaire. Journal of Adolescence.

[bib0036] Prior M., Virasinghe S., Smart D. (2005). Behavioural problems in Sri Lankan schoolchildren. Associations with socio-economic status, age, gender, academic progress, ethnicity and religion. Social Psychiatry and Psychiatric Epidemiology.

[bib0038] Rodríguez-Fernández A., Ramos-Díaz E., Fernández-Zabala A., Goñi E., Esnaola I., Goñi A. (2016). Contextual and psychological variables in a descriptive model of subjective well-being and school engagement. International Journal of Clinical and Health Psychology.

[bib0039] Rodríguez-Hernández P.J., Betancort M., Ramírez-Santana G.M., García R., Sanz-Álvarez E.J., De las Cuevas-Castresana C. (2012). Psychometric properties of the parent and teacher versions of the Strength and Difficulties Questionnaire (SDQ) in a Spanish sample. International Journal of Clinical and Health Psychology.

[bib0040] Ross D.A., Hinton R., Melles-Brewer M., Engel D., Zeck W., Fagan L., Herat J., Phaladi G., Imbago-Jácome D., Anyona P., Sanchez A., Damji N., Terki F., Baltag V., Patton G., Silverman A., Fogstad H., Banerjee A., Mohan A. (2020). Adolescent well-being: A definition and conceptual framework. Journal of Adolescent Health.

[bib0041] Ruchkin V., Koposov R., Schwab-Stone M. (2007). The strength and difficulties questionnaire: Scale validation with Russian adolescents. Journal of Clinical Psychology.

[bib0042] Salmera-Aro, K. (2011). Stages of adolescence. In B. Bradford Brown M. J. Prinstein, Encyclopedia of Adolescence Vol. 1, pp. 360-368). Oxford: Elsevier.

[bib0043] Stone L.L., Janssens J.M.A.M., Vermulst A.A., Van Der Maten M., Engels R.C.M.E., Otten R. (2015). The Strengths and Difficulties Questionnaire: psychometric properties of the parent and teacher version in children aged 4–7. BMC Psychology.

[bib0044] Stone L.L., Otten R., Engels R.C.M.E., Vermulst A.A., Janssens J.M.A.M. (2010). Psychometric properties of the parent and teacher versions of the strengths and difficulties questionnaire for 4- to 12-Year-olds: A review. Clinical Child and Family Psychology Review.

[bib0045] UNICEF (2021). https://www.unicef.org/reports/state-worlds-children-2021.

[bib0046] Van Roy B., Veenstra M., Clench-Aas J. (2008). Construct validity of the five-factor Strengths and Difficulties Questionnaire (SDQ) in pre-, early, and late adolescence. Journal of Child Psychology and Psychiatry, and Allied Disciplines.

[bib0047] Vugteveen J., de Bildt A., Timmerman M.E. (2022). Normative data for the self-reported and parent-reported Strengths and Difficulties Questionnaire (SDQ) for ages 12-17. Child and Adolescent Psychiatry and Mental Health.

[bib0048] Yao S., Zhang C., Zhu X., Jing X., McWhinnie C.M., Abela J.R.Z. (2009). Measuring adolescent psychopathology: psychometric properties of the self-report strengths and difficulties questionnaire in a sample of Chinese adolescents. Journal of Adolescent Health.

