# Supplementary material for: Spanish normative data of the Strengths and Difficulties Questionnaire in a community-based sample of adolescents: Datos normativos españoles del Cuestionario de capacidades y dificultades (SDQ) en una muestra comunitaria de adolescentes
Source: Int J Clin Health Psychol. 2022 Aug 29;22(3):100328. doi: 10.1016/j.ijchp.2022.100328 (PMC9442435; doi:10.1016/j.ijchp.2022.100328)
Supplement: Supplementary file 1 [file mmc1.docx]

**APPENDIX A** Supplementary data

**Table S1.** Descriptive statistics for the SDQ scores

**Table S2.** Internal Consistency for scores of the SDQ scales and items

**Table S3.** Goodness-of- fit indices of the confirmatory factor analysis

**Table S4.** Banding scores for SDQ in younger adolescents (14- to 16-year-olds)

**Table S5.** Banding scores for SDQ in older adolescents (17- to 18-year-olds)

**Table S6.** Banding scores for SDQ in males

**Table S7.** Banding scores for SDQ in females

**Table S1.** Descriptive statistics (mean, standard deviation in parenthesis) for the scores of the Strengths and Difficulties Questionnaire

|  | Gender | | Age | |  |
| --- | --- | --- | --- | --- | --- |
| Subscales | Males  (*n* = 1,561) | Females  (*n* = 1,804) | Younger  (*n* = 2,288) | Older  (n = 1,090) | Total  (*N* = 3,370) |
| Emotional | 2.63 (2.09) | 4.19 (2.44) | 3.42 (2.43) | 3.51 (2.41) | 3.47 (2.42) |
| Conduct | 2.06 (1.73) | 1.71 (1.53) | 1.92 (1.66) | 1.84 (1.61) | 1.87 (1.63) |
| Peer | 1.41 (1.46) | 1.52 (1.66) | 1.41 (1.64) | 1.54 (1.54) | 1.48 (1.59) |
| Hyperactivity | 4.27 (2.22) | 4.41 (2.13) | 4.33 (2.22) | 4.36 (2.15) | 4.34 (2.18) |
| Prosocial | 8.35 (1.54) | 8.74 (1.35) | 8.51 (1.50) | 8.60 (1.42) | 8.56 (1.45) |
| TD | 10.37 (5.11) | 11.83 (5.15) | 11.08 (5.42) | 11.25 (5.02) | 11.17 (5.19) |

*Note*. TD = Total Difficulties.

**Table S2.** Internal Consistency (McDonald’s Omega) for scores of the SDQ subscales and items

| Subscales and items | Internal Consistency *(ω)* | 95% lower and upper bounds |  |
| --- | --- | --- | --- |
| **Emotional** | .71 | .70-.73 |  |
| If item 3 deleted | .70 | .68-.71 |  |
| If item 8 deleted | .66 | .64-.67 |  |
| If item 13 deleted | .62 | .60-.65 |  |
| If item 16 deleted | .67 | .65-.68 |  |
| If item 24 deleted | .67 | .66-.69 |  |
| **Conduct** | .51 | .48-.54 |  |
| If item 5 deleted | .45 | .42-.48 |  |
| If item 7 deleted | .46 | .43-.49 |  |
| If item 12 deleted | .46 | .43-.49 |  |
| If item 18 deleted | .45 | .42-.49 |  |
| If item 22 deleted | .47 | .44-.50 |  |
| **Peer** | .58 | .56-.60 |  |
| If item 6 deleted | .49 | .47-.52 |  |
| If item 11 deleted | .55 | .53-.58 |  |
| If item 14 deleted | .51 | .48-.54 |  |
| If item 19 deleted | .54 | .51-.56 |  |
| If item 23 deleted | .55 | .52-.57 |  |
| **Hyperactivity** | .63 | .61-.65 |  |
| If item 2 deleted | .58 | .56-.61 |  |
| If item 10 deleted | .60 | .58-.62 |  |
| If item 15 deleted | .64 | .62-.66 |  |
| If item 21 deleted | .64 | .62-.66 |  |
| If item 25 deleted | .66 | .65-.68 |  |
| **Prosocial** | .57 | .54-.59 |  |
| If item 1 deleted | .51 | .48-.54 |  |
| If item 4 deleted | .53 | .51-.56 |  |
| If item 9 deleted | .48 | .45-.51 |  |
| If item 17 deleted | .54 | .51-.56 |  |
| If item 20 deleted | .50 | .47-.52 |  |

**Table S3.** Goodness-of- fit indices of the confirmatory factor analysis

| Models | *χ2* | *df* | CFI | TLI | RMSEA  (CI 90%) | SRMR |
| --- | --- | --- | --- | --- | --- | --- |
| (a) Baseline three-factor | 7918.82 | 272 | .63 | .59 | .091 (.090-.093) | .09 |
| (b) Three-factor with CE | 4334.82 | 267 | .80 | .78 | .067 (.065-.069) | .07 |
| (c) Baseline five-factor | 4095.90 | 265 | .81 | .79 | .065 (.064-.067) | .08 |
| (d) Five-factor with CE | 1708.58 | 262 | .91 | .90 | .041 (.039-.042) | .04 |
| (e) Second-order factor | 4864.69 | 268 | .78 | .75 | .071 (.070-.073) | .08 |
| (f) Second-order factor with CE | 2966.66 | 263 | .87 | .85 | .055 (.053-.057) | .06 |
| (g) Bifactor model | 3482.90 | 249 | .90 | .91 | .052 (.050-.054) | .05 |
| (h) Bifactor model with CE | 2731.78 | 245 | .92 | .91 | .057 (.055-.059) | .04 |

*Note*. χ^2^ = Chi square; *df* = degrees of freedom; CFI = Comparative Fit Index; TLI = Tucker-Lewis Index; RMSEA = Root Mean Square Error of Approximation; CI = Confidence Interval; SRMR = Standardized Root Mean Square Residual; CE = Correlated Errors.

**Table S4.** Banding scores for the Strengths and Difficulties Total difficulties score and subscales in younger adolescents (14- to 16-year-olds, n = 2,288)

|  | **Total** | | | **Emotional** | | | **Conduct** | | | **Hyper** | | **Peer** | | | **Prosocial** | | |  |
| --- | --- | --- | --- | --- | --- | --- | --- | --- | --- | --- | --- | --- | --- | --- | --- | --- | --- | --- |
|  | **Raw** | **PR** | | **Raw** | **PR** | | **Raw** | **PR** | | **Raw** | **PR** | **Raw** | | **PR** | **Raw** | | **PR** |  |
|  | 0-8 | <35 | | 0 | 11 | | 0 | 21 | | 0 | 3 | 0 | | 34 | 0-3 | | 0 |  |
|  | 9 | 35 | | 1 | 12 | | 1 | 22 | | 1 | 4 | 1 | | 35 | 4 | | 1 |  |
|  | 10 | 43 | | 2 | 27 | | 2 | 48 | | 2 | 11 | 2 | | 63 | 5 | | 2 |  |
|  | 11 | 51 | | 3 | 43 | | 3 | 70 | | 3 | 22 | 3 | | 82 | 6 | | 5 |  |
|  | 12 | 58 | | 4 | 58 | | 4 | 85 | | 4 | 38 | 4 | | 90 | 7 | | 10 |  |
|  | 13 | 65 | | 5 | 71 | | 5 | 93 | | 5 | 54 | 5 | | 95 | 8 | | 22 |  |
|  | 14 | 71 | | 6 | 81 | | 6 | 98 | | 6 | 71 | 6 | | 98 | 9 | | 42 |  |
|  | 15 | 78 | | 7 | 89 | | 7 | 99 | | 7 | 84 | 7 | | 99 | 10 | | 70 |  |
|  | 16 | 82 | | 8 | 95 | | 8-10 | 100 | | 8 | 92 | 8-10 | | 100 |  | |  |  |
|  | 17 | 86 | | 9 | 98 | |  |  | | 9 | 98 |  | |  |  | |  |  |
|  | 18 | 90 | | 10 | >99 | |  |  | | 10 | 100 |  | |  |  | |  |  |
|  | 19 | 92 | |  |  | |  |  | |  |  |  | |  |  | |  |  |
|  | 20 | 94 | |  |  | |  |  | |  |  |  | |  |  | |  |  |
|  | 21 | 96 | |  |  | |  |  | |  |  |  | |  |  | |  |  |
|  | 22 | 97 | |  |  | |  |  | |  |  |  | |  |  | |  |  |
|  | 23-24 | 98 | |  |  | |  |  | |  |  |  | |  |  | |  |  |
|  | 25 | 99 | |  |  | |  |  | |  |  |  | |  |  | |  |  |
|  | 26-33 | 100 | |  |  | |  |  | |  |  |  | |  |  | |  |  |
| **Banding ranges (%)** | | | | | | | | | | | | | | | | | | |
| NC | 0-15 (81.2) | | 0-5 (80.3) | | | 0-3 (84.3) | | | 0-6 (83.6) | | | | 0-2 (81.0) | | | 8-10 (78.6) | |  |
| AR | 16-17 (7.8) | | 6-7 (13.7) | | | 4 (8.1) | | | 7 (8.3) | | | | 3 (9.0) | | | 7 (12.0) | |  |
| CL | 18-40 (11) | | 8-10 (6.0) | | | 5-10 (7.6) | | | 8-10 (8.1) | | | | 4-10 (10.0) | | | 0-6 (9.4) | |  |

*Note*. Hyper = Hyperactivity; Raw = Raw Score; PR = Percentile; NC = Non-Clinical; AR = At Risk; CL = Clinical.

**Table S5.** Banding scores for the Strengths and Difficulties Total difficulties score and subscales in older adolescents (17- to 18-year-olds, n = 1,090)

|  | **Total** | | | **Emotional** | | | **Conduct** | | | **Hyper** | | **Peer** | | | **Prosocial** | | |
| --- | --- | --- | --- | --- | --- | --- | --- | --- | --- | --- | --- | --- | --- | --- | --- | --- | --- |
|  | Raw | PR | | Raw | PR | | Raw | PR | | Raw | PR | Raw | | PR | Raw | | PR |
|  | 0-8 | <29 | | 0 | 7 | | 0 | 22 | | 0 | 3 | 0 | | 26 | 0-3 | | 0 |
|  | 9 | 29 | | 1 | 8 | | 1 | 23 | | 1 | 4 | 1 | | 27 | 4 | | 1 |
|  | 10 | 38 | | 2 | 20 | | 2 | 49 | | 2 | 10 | 2 | | 58 | 5 | | 2 |
|  | 11 | 46 | | 3 | 37 | | 3 | 72 | | 3 | 20 | 3 | | 78 | 6 | | 4 |
|  | 12 | 52 | | 4 | 52 | | 4 | 87 | | 4 | 36 | 4 | | 89 | 7 | | 9 |
|  | 13 | 60 | | 5 | 65 | | 5 | 94 | | 5 | 52 | 5 | | 95 | 8 | | 18 |
|  | 14 | 68 | | 6 | 75 | | 6 | 97 | | 6 | 69 | 6 | | 98 | 9 | | 37 |
|  | 15 | 73 | | 7 | 85 | | 7 | 99 | | 7 | 83 | 7-10 | | 100 | 10 | | 67 |
|  | 16 | 80 | | 8 | 93 | | 8-10 | 100 | | 8 | 93 |  | |  |  | |  |
|  | 17 | 84 | | 9 | 97 | |  |  | | 9 | 98 |  | |  |  | |  |
|  | 18 | 87 | | 10 | 100 | |  |  | | 10 | 100 |  | |  |  | |  |
|  | 19 | 91 | |  |  | |  |  | |  |  |  | |  |  | |  |
|  | 20 | 94 | |  |  | |  |  | |  |  |  | |  |  | |  |
|  | 21-22 | 96 | |  |  | |  |  | |  |  |  | |  |  | |  |
|  | 23-24 | 98 | |  |  | |  |  | |  |  |  | |  |  | |  |
|  | 25 | 99 | |  |  | |  |  | |  |  |  | |  |  | |  |
|  | 26-33 | 100 | |  |  | |  |  | |  |  |  | |  |  | |  |
| **Banding ranges (%)** | | | | | | | | | | | | | | | | | |
| NC | 0-16 (83.3) | | 0-6 (84.5) | | | 0-3 (86.0) | | | 0-6 (82.2) | | | | 0-3 (88.6) | | | 8-10 (82.2) | |
| AR | 17-18 (7.6) | | 7 (7.6) | | | 4 (7.7) | | | 7 (9.8) | | | | 4 (5.8) | | | 7 (9.3) | |
| CL | 19-40 (9.1) | | 8-10 (7.9) | | | 5-10 (6.3) | | | 8-10 (8.0) | | | | 5-10 (5.6) | | | 0-6 (8.5) | |

*Note*. Hyper = Hyperactivity; Raw = Raw Score; PR = Percentile; NC = Non-Clinical; AR = At Risk; CL = Clinical.

**Table S6.** Banding scores for the Strengths and Difficulties Total difficulties score and subscales in males (n = 1,561)

|  | **Total** | | | **Emotional** | | | **Conduct** | | **Hyper** | | | **Peer** | | | | **Prosocial** | |
| --- | --- | --- | --- | --- | --- | --- | --- | --- | --- | --- | --- | --- | --- | --- | --- | --- | --- |
|  | Raw | PR | | Raw | PR | | Raw | PR | Raw | | PR | Raw | | PR | | Raw | PR |
|  | 0-8 | <40 | | 0 | 16 | | 0 | 19 | 0 | | 4 | 0 | | 31 | | 0-2 | 0 |
|  | 9 | 40 | | 1 | 34 | | 1 | 20 | 1 | | 5 | 1 | | 32 | | 3 | 1 |
|  | 10 | 48 | | 2 | 35 | | 2 | 44 | 2 | | 12 | 2 | | 63 | | 4 | 2 |
|  | 11 | 55 | | 3 | 56 | | 3 | 67 | 3 | | 23 | 3 | | 81 | | 5 | 3 |
|  | 12 | 62 | | 4 | 71 | | 4 | 82 | 4 | | 39 | 4 | | 91 | | 6 | 5 |
|  | 13 | 69 | | 5 | 82 | | 5 | 91 | 5 | | 55 | 5 | | 96 | | 7 | 12 |
|  | 14 | 75 | | 6 | 90 | | 6 | 96 | 6 | | 72 | 6 | | 99 | | 8 | 26 |
|  | 15 | 80 | | 7 | 95 | | 7 | 99 | 7 | | 84 | 7-10 | | 100 | | 9 | 47 |
|  | 16 | 85 | | 8 | 98 | | 8-10 | 100 | 8 | | 92 |  | |  | | 10 | 73 |
|  | 17 | 88 | | 9 | 99 | |  |  | 9 | | 98 |  | |  | |  |  |
|  | 18 | 91 | | 10 | 100 | |  |  | 10 | | 100 |  | |  | |  |  |
|  | 19 | 93 | |  |  | |  |  |  | |  |  | |  | |  |  |
|  | 20 | 95 | |  |  | |  |  |  | |  |  | |  | |  |  |
|  | 21 | 97 | |  |  | |  |  |  | |  |  | |  | |  |  |
|  | 22-23 | 98 | |  |  | |  |  |  | |  |  | |  | |  |  |
|  | 24 | 99 | |  |  | |  |  |  | |  |  | |  | |  |  |
|  | 25-40 | 100 | |  |  | |  |  |  | |  |  | |  | |  |  |
| **Banding ranges (%)** | | | | | | | | | | | | | | | | | |
| NC | 0-15 (84.5) | | 0-4 (81.5) | | | 0-3 (81.8) | | | | 0-6 (83.6) | | | 0-2 (80.9) | | 8-10 (74.6) | | |
| AR | 16-17 (6.28) | | 5 (7.8) | | | 4 (8.5) | | | | 7 (7.9) | | | 3 (9.8) | | 7 (14.1) | | |
| CL | 18-40 (9.22) | | 6-10 (10.7) | | | 5-10 (9.7) | | | | 8-10 (8.5) | | | 4-10 (9.3) | | 0-6 (11.3) | | |

*Note*. Hyper = Hyperactivity; Raw = Raw Score; PR = Percentile; NC = Non-Clinical; AR = At Risk; CL = Clinical.

**Table S7.** Banding scores for the Strengths and Difficulties Total difficulties score and subscales in females (n = 1,804)

|  | **Total** | | **Emotional** | | | **Conduct** | | | **Hyper** | | | | **Peer** | | | **Prosocial** | |
| --- | --- | --- | --- | --- | --- | --- | --- | --- | --- | --- | --- | --- | --- | --- | --- | --- | --- |
|  | Raw | PR | | Raw | PR | | Raw | PR | | Raw | PR | Raw | | PR | Raw | | PR |
|  | 0-8 | <27 | | 0 | 5 | | 0 | 24 | | 0 | 2 | 0 | | 32 | 0-3 | | 0 |
|  | 9 | 27 | | 1 | 6 | | 1 | 25 | | 1 | 3 | 1 | | 33 | 4 | | 1 |
|  | 10 | 36 | | 2 | 15 | | 2 | 52 | | 2 | 10 | 2 | | 61 | 5 | | 2 |
|  | 11 | 44 | | 3 | 28 | | 3 | 74 | | 3 | 20 | 3 | | 79 | 6 | | 3 |
|  | 12 | 52 | | 4 | 43 | | 4 | 88 | | 4 | 35 | 4 | | 89 | 7 | | 8 |
|  | 13 | 58 | | 5 | 58 | | 5 | 96 | | 5 | 52 | 5 | | 94 | 8 | | 16 |
|  | 14 | 65 | | 6 | 70 | | 6-8 | 99 | | 6 | 69 | 6 | | 97 | 9 | | 34 |
|  | 15 | 73 | | 7 | 81 | | 9-10 | 100 | | 7 | 83 | 7 | | 98 | 10 | | 65 |
|  | 16 | 78 | | 8 | 90 | |  |  | | 8 | 93 | 8-10 | | 100 |  | |  |
|  | 17 | 83 | | 9 | 96 | |  |  | | 9 | 98 |  | |  |  | |  |
|  | 18 | 87 | | 10 | >99 | |  |  | | 10 | 100 |  | |  |  | |  |
|  | 19 | 90 | |  |  | |  |  | |  |  |  | |  |  | |  |
|  | 20 | 93 | |  |  | |  |  | |  |  |  | |  |  | |  |
|  | 21 | 95 | |  |  | |  |  | |  |  |  | |  |  | |  |
|  | 22 | 96 | |  |  | |  |  | |  |  |  | |  |  | |  |
|  | 23 | 97 | |  |  | |  |  | |  |  |  | |  |  | |  |
|  | 24-25 | 98 | |  |  | |  |  | |  |  |  | |  |  | |  |
|  | 26 | 99 | |  |  | |  |  | |  |  |  | |  |  | |  |
|  | 27-33 | 100 | |  |  | |  |  | |  |  |  | |  |  | |  |
| **Banding ranges (%)** | | | | | | | | | | | | | | | | | |
| NC | 0-16 (82.2) | | 0-6 (80.9) | | | 0-3 (87.5) | | | 0-6 (82.8) | | | | 0-3 (88.5) | | | 8-10 (84.2) | |
| AR | 17-18 (7.4) | | 7 (8.8) | | | 4 (7.5) | | | 7 (9.5) | | | | 4 (5.3) | | | 7 (8.6) | |
| CL | 19-40 (10.4) | | 8-10 (10.3) | | | 5-10 (5.0) | | | 8-10 (7.7) | | | | 5-10 (6.2) | | | 0-6 (7.2) | |

*Note*. Hyper = Hyperactivity; Raw = Raw Score; PR = Percentile; NC = Non-Clinical; AR = At Risk; CL = Clinical.
